# Food insecurity and its association with socio-demographic characteristics in Cyprus

**DOI:** 10.1017/jns.2024.47

**Published:** 2024-09-19

**Authors:** Maria Kantilafti, Mary Yannakoulia, Konstantinos Giannakou, Maria Kyprianidou, Stavri Chrysostomou

**Affiliations:** 1 Department of Health Sciences, School of Sciences, European University Cyprus, 6 Diogenes Str. Engomi, 2404, P.O. Box: 22006, Nicosia 1516, Cyprus; 2 Department of Nutrition and Dietetics, School of Health Sciences and Education, Harokopio University Athens, Eleftheriou Venizelou Ave.,70, 176 76, Kallithea, Athens, Greece; 3 Department of Life Sciences, School of Sciences, European University Cyprus, 6 Diogenes Str. Engomi, 2404, P.O. Box: 22006, 1516, Nicosia, Cyprus

**Keywords:** Cross-sectional study, Food insecurity, Prevalence, Socio-demographic factors

## Abstract

The prevalence of food insecurity in Cyprus and the socio-demographic factors that are related to this public health problem are unknown. Data used in this cross-sectional study were collected between 1 June 2022 and 21 May 2023 using a self-reported method. Food insecurity was evaluated using the Greek version of Adult Food Security Survey Module. The data regarding socio-demographic and socio-economic characteristics along with body weight and height measurements were collected through self-administered questionnaire. A representative sample of n=1255 adults, ≥18 years old living in the five different districts of the Republic of Cyprus, was recruited. Prevalence of food insecurity in Cypriot population was 12.6%. Prevalence was higher in females, in older adults, in adults living in Paphos, in individuals who were separated, divorced, or widowed, in retired people, in people living with children, and in people with low income and education. Based on multivariable analysis, income was the strongest socio-demographic factor independently associated with food security (€ 6,500–€ 19,500: AOR: 0.49, 95% CI 0.28, 0.86 and >€ 19,500: AOR: 0.15, 95% CI 0.73, 0.31). Food insecurity is a global problem that need further examination. The association between food insecurity and socio-demographic characteristics needs to be highlighted in order for each country to develop specific public health policies (e.g. financial support to low income people) to decrease food insecurity and improve people’s overall health and quality of life.

## Introduction

Food security exists when all people, regardless of season, have physical, social and economic access to safe and nutritious food to meet their needs for an active and healthy life.^(1)^ Based on the above, food insecurity can be defined as limited or uncertain availability of nutritionally adequate and safe foods, or inability to acquire acceptable food in a socially acceptable way.^(2)^

According to the World Food Program, 349 million people in 79 countries are experience acute food insecurity, an increase of 200 million people compared to 2019, before the pandemic of Covid 19.^(3)^ Although food insecurity is generally associated with lower income^(3)^ and it is more prevalent in lower and middle income countries,^(4)^ recent studies revealed that food insecurity is also a concerning public health problem in high income countries.^(5,6)^ In the USA, in 2021, 10.2% of households experienced food insecurity, with 38% of them experiencing severe food insecurity.^(7)^ In regards to Canada, in 2019, 14.2% of women and 12.1% of men experienced food insecurity.^(8)^ As for the European countries, in Denmark, in 2015, the prevalence of low and very low food security was 6% and 2.4% respectively.^(9)^ A recent study conducted in Finland concluded that 65% of private sector service workers were food insecure with over a third (36%) reposting severe food insecure.^(10)^ The data regarding the prevalence of food insecurity in the Mediterranean countries are scarce. Bocquier et al, reported that, in 2015, 12.2% of French adults citizens were food insecure.^(11)^ As for Greece, two cross-sectional studies were conducted in 2016 among Greek university students^(12)^ and in 2019 among older adults.^(13)^ Theodoridis et al, concluded that a significant high proportion of Greek university students were severe food insecure (45.3%) and 22.5% and 14.8% of the sample were moderate and low food insecure.^(12)^ The study by Gkiouras et al highlights the high prevalence (50.4%) of food insecurity in older adults.^(13)^ The only study occurred in Cyprus aiming to examine the risk of experiencing food stress, defined as the product of the cost of healthy food relative to the income of the household, among the low-income Cypriot population was conducted by Chrysostomou et al, in 2017.^(14)^ The researchers concluded that there is a significant proportion of low income Cypriots who experience food stress. There are no available data in regards to the prevalence of food insecurity in the general population in Cyprus.

Socio-demographic factors such as age, sex, province and territory, education, household structure and socio-economic factor such as income are a group of variables that affect food insecurity.^(15–17)^ It has been found that, there is an inverse association between income and food insecurity. Specifically, for every decrease in a family income unit, the food insecurity increases by 12.5%.^(18)^ Baxter et al, stated that people who receive less than €20 000 annually are at greater risk for food insecurity compared with those who receive more than €20 000 annually.^(16)^ Moreover, a recent cross-sectional study conducted in USA, by Cai et al, analyzed data from the «National Health Interview Survey- NHIS» (2020–2021) and they concluded that young people, female sex, certain nationalities, being unmarried, divorced or widowed, having low education and no health insurance were significantly associated with food insecurity.^(17)^ In addition, the odds of being food insecure were higher for unmarried volunteers and for households with children in the house.^(15)^

In a context of continuously rising food costs, there is a general perception that healthy eating is more expensive compared to unhealthy eating.^(18)^ People experiencing food insecurity, due to the lack of money, are forced to consume energy-dense foods and foods high in sugar and fat^(19)^ which are cheaper compared to foods that promote a healthy nutritional behavior^(20)^ such as fruits, vegetables, and dairy products.^(21)^ A recent study, conducted in Cyprus, concluded that low-income people tend to spent almost two third of their monthly income in order to buy nutritious foods and therefore, low-income people in Cyprus are food insecure.^(14)^ Due to the consumption of unhealthy foods, food insecure people are more likely to suffer from chronic diseases such as diabetes, cardiovascular diseases and hypertension^(22)^ compared with food secure people. Other chronic health conditions associated with food insecurity are anemia, especially in women,^(20)^ depression,^(23)^ and coeliac disease.^(24)^

Notably, the prevalence of food insecurity among the whole population in Cyprus has not yet been evaluated. Moreover, the factors affecting food insecurity have not been well established considering that the risk of poverty in Cyprus, even though it records a slight download course, it nevertheless remains at high levels compared with other European countries such as Slovakia, Finland, Poland, Slovenia, and Czechia.^(25)^ Based on the latest data, in 2021, the risk of poverty in Cyprus was 13.8%, compared to 15.9% in 2008.^(26)^ Considering that poverty is only one factor associated with food insecurity, other factors may also play an important role in this phenomenon.^(15–17)^ Therefore, the aim of the study was to assess the prevalence of food insecurity among Cypriot population and its relationship with socio-demographic factors.

## Methods

This study used a community-based cross-sectional study design. A total of 1255 men and women >18 years’ old living in the five different districts of the Republic of Cyprus participated in the study. Data used in this cross sectional study were collected between 1 June 2022 and 21 May 2023 to assess the prevalence of food insecurity and associated factors including socio-demographic and socio-economic factors in a nationally representative sample of the Cypriot population in terms of region, age and sex.

The required sample size for the estimation of the prevalence of food insecurity was 1255 individuals with a precision of 2%, using a 95% confidence interval (CI) and assuming a true prevalence of food insecurity to be 12%.

In order to achieve a representative sample of the Cypriot population, using the quote sampling method, the sample was divided into the five municipalities of Cyprus (Nicosia, Larnaca, Limassol, Ammochostos, and Paphos) based on the latest available data (2011). Afterwards, the sample was further divided in regards to sex and age creating four age groups (18–24, 25–44, 45–64, and ≥65 years old).^(22)^ The sample nationally representativeness was confirmed using the Chi-square goodness of fit test with statistical significant to be <0.05. Recruitment occurred in public areas (e.g. malls, supermarkets, and churches).

After obtaining the consent form, participants completed a standardised questionnaire^(27)^ and a questionnaire that has been used in previous studies in the Cypriot population.^(22,28)^ In case of participant’s inability to complete the questionnaire, two trained researchers were present to offer any help needed. The first questionnaire included close ended questions about socio-demographic characteristics (i.e. age, sex, body weight, height, body mass index-BMI, marital status, employment, income, education, and family composition) where the second questionnaire was about the assessment of food security.

In regards to socio-demographic characteristics, age, height, and body weight were self-reported. BMI was derived from a ratio of weight in kilograms divided by height in meter squared. The three BMI groups were defined based on the National Heart, Lung and Blood Institute terminology (underweight<18.5, normal weight: 18.5–<25, overweight: 25–<30 and obese:≥30).^(29)^ Marital status was recorded as never married, married, or living with partner and separated, divorced, or widowed. Employment was classified into three categories: employed, unemployed, and retired. In regards to income, as a socio-economic factor, three categories were used: low (€6,500), moderate, (€6,500–19,500), and high (*>*€19,500). Primary (<7 years of schooling), secondary (7–12 years of schooling), and higher (>2 years of schooling) education were used to evaluated participant’s education. For the evaluation of family composition, participants reported the number of people living in the same house with them and how many of them are under 18 or over 75 years old. The same questionnaire had been used in previous studies conducted in Cyprus.^(22,28)^

The outcome variable for this study was food insecurity which was evaluated using the Greek version of Adult Food Security Survey Module (AFSSM).^(27)^ The above-mentioned questionnaire is a self-reported questionnaire which evaluates adult’s food insecurity status over the past 12 months. It consists of 10 close ended questions and can be used for households with or without children. The questions are regarding food-related behaviors, experiences, and conditions that are common in people experiencing food insecurity. Affirmative responses to three or more items indicated that that the participant experienced food insecurity.^(30)^ Based on the study conducted in Greece, by Michalis et al, the Cronbach’s alpha coefficient of reliability for the Greek AFSSM was good (0.763 and 0.783, respectively, for the 2 measurements).^(27)^

During the study, participants had the opportunity to be informed regarding the purpose of the study as well as the method of data collection and processing by two skilled researchers in public areas, including but not limited to shopping centres, malls, churches, and universities. The participants were also informed that the above study was conducted anonymously and that the latter was also approved by the local Bioethics Committee. Lastly, it is noted that all participants provided relevant consent for their participation prior the provision of the questionnaire.

Kolmogorov–Smirnoff test of normality was used to assess the distribution of age and BMI Descriptive analysis was done to assess the prevalence of food insecurity. The continuous variables (e.g. age and BMI) are presented as mean ± standard deviation (SD) and categorical variables (e.g. age group, sex, BMI group, marital status, employment, income, and education) as absolute and relative (%) frequencies. Baseline characteristics of participants by food insecurity group were examined using Pearson’s chi-square test for categorical variables and Independent Sample t-test for continuous variables. At first, univariate analysis was performed in order to assess the relationship between food insecurity (which was defined as the outcome of measure) and each socio-demographic and socio-economic factor separately (which were defined as the exposures) (model 1). Afterwards, we performed a multivariate analysis (model 2) in order to assess the association between food insecurity and each socio-demographic factor controlling for the other socio-demographic factors (such as age, employment, marital status living with persons < 18 years old, income, and education). Statistical analysis was conducted using SPSS v.29.0 with statistical significance level set at P < 0.05.

## Results

Table 1 shows the socio-demographic and socio-economic characteristics of participants by food security status. The study consisted of 1255 people, men and women, with mean age 43.43 (SD ± 17.07) years. More than a half of the volunteers were females (54.4%), 48.7% were living with a child and 10.4% were living with an older adult (>75 years). Furthermore, the majority of the sample (26.3%) was living in a household with 3 persons. Moreover, 46.0% had a normal body weight, according to their BMI, whereas 33.8% and 17.1 % were overweight and obese respectively. In regards to region, most of the participants were living in Nicosia (34.7%) and Larnaca (27.5%) and the rest of them were living in Limassol (23.6%), Paphos (8.9%) and Ammochostos (5.3%). Over a third of the sample were married (66.0%), 70.6% were employed and 17.7% of the participants had an annual income < €6,500. As for education, the majority of the sample (68.7%) reported higher education.

**Table 1.** Socio-demographic characteristics overall and by food insecurity group

	Total^a^ (n = 1255)	Food insecurity^b^ (n = 229)	Food security^b^ (n = 1026)	*P* value
Age (years)^*^	43.43 ± 17.07	46.46 ± 19.48	42.99 ± 16.66	**0.017** ^c^
Age group				**<0.001** ^d^
18–24	194 (15.5)	27 (13.9)	167 (86.1)	
25–44	507 (40.4)	47 (9.3)	460 (90.7)	
45–64	357 (28.4)	42 (11.8)	315 (88.2)	
≥65	197 (15.7)	42 (21.3)	155 (78.7)	
Gender				0.348^d^
Male	572 (45.6)	78 (13.6)	494 (86.4)	
Female	683 (54.4)	80 (11.7)	603 (88.3)	
BMI (kg/m^2^)	25.86 ± 5.29	27.00 ± 5.64	25.63 ± 5.39	**0.03** ^c^
BMI group (kg/m^2^)				0.082^d^
Underweight (<18.5)	40 (3.2)	5 (12.5)	35 (87.5)	
Normal weight (18.5–24.9)	577 (46.0)	63 (10.9)	514 (89.1)	
Overweight (25–29.9)	424 (33.8)	52 (12.3)	372 (87.7)	
Obese (≥30)	214 (17.1)	38 (17.8)	176 (82.2)	
Region				**0.031** ^d^
Ammochostos	66 (5.3)	8 (12.1)	58(87.9)	
Larnaca	345 (27.5)	35 (10.1)	310(89.9)	
Limassol	296 (27.5)	33 (11.1)	263(88.9)	
Nicosia	436 (27.5)	58 (13.3)	378(86.7)	
Paphos	112 (27.5)	24 (21.4)	88 (78.6)	
Marital status				**<0.001** ^d^
Single	339 (27.1)	43 (12.7)	296 (87.3)	
Married or living with partner	825 (66.0)	92 (11.2)	733 (88.8)	
Separated, divorced, or widowed	86 (6.9)	23 (26.7)	63 (73.3)	
Individuals in the family				0.442^d^
1	107 (8.5)	18 (16.8)	89 (83.2)	
2	325 (25.9)	45 (13.8)	280 (86.2)	
3	255 (20.3)	27 (10.6)	228 (89.4)	
4	330 (26.3)	42 (10.9)	288 (87.3)	
≥5	238 (19.0)	26 (10.9)	212 (89.1)	
Persons < 18 years old				**0.009** ^**d**^
0	644 (51.3)	97 (10.9)	547 (84.9)	
≥1	611 (48.7)	64 (10.9)	550 (90.0)	
Persons ≥ 75 years old				0.779 ^d^
0	1124 (89.6)	140 (12.5)	984 (87.5)	
≥1	131 (10.4)	18 (13.7)	113 (86.3)	
Employment				**<0.001** ^d^
Employed	883 (70.6)	84 (9.5)	799 (90.5)	
Unemployed	198 (15.8)	34 (17.2)	164 (82.8)	
Retired	170 (13.6)	39 (22.9)	131 (77.1)	
Income				**<0.001** ^d^
<€ 6,500	206 (17.7)	51 (24.8)	155 (75.2)	
€ 6,500–€ 19,500	517 (44.3)	74 (14.3)	443 (85.7)	
>€ 19,500	444 (38.0)	20 (4.5)	424 (95.5)	
Education				**<0.001** ^**d**^
Primary	77 (6.2)	20 (26.0)	57 (74.0)	
Secondary	314 (25.1)	56 (17.8)	258 (82.2)	
Higher	859 (68.7)	82 (9.5)	777 (90.5)	

Abbreviations: BMI, body mass index.

^a^Means ± sd, ^b^n (%), ^c^Independent sample t-test, ^d^Pearson’s chi-squared test.

* Bold values represent statistically significant associations P < 0.005.

Our results indicate that 12.6% of the volunteers are food insecure. Regarding the socio-demographic and socio-economic characteristics our findings, show that age, BMI, region, marital status, living with persons < 18 years old, employment status, income, and education were all significantly associated with food insecurity (P < 0.05). Moreover, among individuals with food insecurity, participants were older (21.3%), living in Paphos (21.3%), separated, divorced or widowed (26.7%), retired (22.9%), living with no children (15.1%), having an income less than €6,500, and report low education (26.0%) compared to food secure participants. Also, participants with food insecurity had a higher BMI compared to those without experiencing food insecurity (27.00 ± 5.64 vs 25.63 ± 5.39) (Table 1).

Logistic regression analysis was conducted in order to examine the relation between food insecurity and socio-demographic and socio-economic characteristics (Table 2). The main finding from the adjusted model indicates that people with income € 6,500–€ 19,500 and >€ 19,500 had 50.6% (AOR: 0.49, 95% CI 0.28,0.86) and 85.1% (AOR: 0.15, 95% CI 0.73,0.31) lower risk for food insecurity compared to participants with income < € 6,500. As for the unadjusted model, being a separated, divorced or widowed increased the risk for food insecurity by 2.5 times (OR: 2.51, 95% CI 1.41, 4.47) compared to people who never got married. Furthermore, individuals who are unemployed or retired had 1.97 (OR: 1.97 [95%CI: 1.28, 3.04]) and 2.83 (OR: 2.83 [95%CI: 1.06, 1.32]) higher risk to present food insecurity, respectively, compared to those who are employed. Moreover, it seems that participants who had higher education presented lower risk for food insecurity OR: 0.30 [95% CI: 0.17, 0.53] compared with people having a primary education. The same relationship seems to apply for participants who were living with no children. More specifically, participants living with no children presented lower risk for food insecurity OR: 0.63 [95%CI: 0.05, 0.88] compared with participants who were living in the same house with children.

**Table 2.** Logistic regression analysis to evaluate the association of socio-demographic characteristics with food insecurity

	Total (*n* = 1255)	
	Model 1^a^	Model 2^b^
Age group		
18–24	1.00	1.00
25–44	0.63 (0.381, 1.05)	1.18 (0.59, 2.37)
45–64	0.823 (0.491, 1.39)	1.38 (0.63, 3.15)
≥65	1.68 (0.986, 2.85)	0.81 (0.26, 2.50)
Persons < 18 years old (0/≥ 1)	**0.63 (0.45**, 0.88)*	0.92 (0.61, 1.39)
Employment		
Employed	1.00	1.00
Unemployed	1.97 (1.28, 3.04)*	1.09 (0.55, 2.19)
Retired	2.83 (1.06, 1.32)*	1.69 (0.67, 4.17)
Marital status		
Never married	1.00	1.00
Married or living with partner	0.86 (0.59, 1.27)	0.93 (0.51, 1.67)
Separated, divorced, or Widowed	**2.51 (1.41**, 4.47)*	1.93 (0.85, 4.35)
Income		
<€ 6,500	1.00	1.00
€ 6,500–€ 19,500	**0.51 (0.34**, 0.76)*	0.49 (0.28, 0.86)*
>€ 19,500	**0.14 (0.08**, 0.25)*	0.15 (0.73, 0.31)*
Education		
Primary	1.00	1.00
Secondary	0.62 (0.34, 1.11)	1.37 (0.65, 2.91)
Higher	**0.30 (0.17**, **0.53)**	1.002 (0.44, 2.29)

^a^univariate analysis, ^b^multivariate analysis.

* Bold values represent statistically significant associations *P* < 0.001.

## Discussion

To the best of our knowledge, this is the first study in Cyprus to assess the prevalence of food insecurity and examine the effect of socio-demographic characteristics on food insecurity in a representative sample of adult population. The prevalence of food insecurity in Cyprus is 12.6%. Among socio-demographic and socio-economic characteristics, income has the strongest relationship with food insecurity whereas all the other characteristics were not significantly related.

In regards to the prevalence of food insecurity in Cyprus, we found that it’s similar to other European countries such as France (12.2%)^(11)^ and Portugal (16.5%).^(31)^ In the present study, income was the strongest socio-economic factor independently associated with food security. Similar results were shown from other researchers,^(31)^ were annual income was found to be an independent factor affecting food insecurity. A recent systematic review, about food insecurity and related contributing factors demonstrated that various socio-demographic characteristics including income influence food insecurity in older adults.^(32)^ Furthermore, Nagata et al, performed an analysis using data from U.S. Census Household Pulse Survey and concluded that income below the federal line is associated with food insecurity.^(33)^ No other socio-demographic factor has been found to have statistically significant effect on food insecurity after controlling for the rest of socio-demographic factors (i.e. age, BMI, region, marital status, living with persons < 18 years old, employment status, and education). A finding which agrees with ours. Another study in USA indicated that from a group of socio-demographic characteristics (i.e. age, sex, race, household income, employment, children in home, and married) only income, marriage and living with children were significantly associated with food insecurity.^(15)^ In contrast, previous studies conducted in USA,^(16,17)^ indicated that individuals with low education are more common to present food insecurity compared with individuals without food insecurity. Similarly, other studies also found that the prevalence of food insecurity increase with low education, living with children under 18, low income, pension, female sex, larger family size, being widowed.^(18,32)^ Therefore, it seems that for other countries characteristics other than income are associated with food insecurity.

Social, cultural and economic differences across countries may explain the above findings. The FAO worldwide study of 147 countries^(34)^ aimed to compare food insecurity in different subpopulations across countries and to assess which factors affect food insecurity and concluded that different socio-demographic may affect food insecurity in each country. For example, in developed countries, key determinant of food insecurity is having a lower level of education which is often related to having no decent job. However, among less developed countries, sex appears to have a significant impact on food insecurity and more particular, women are in a much higher risk of developing food insecurity compared to men^(35)^ which comes in line with other studies.^(18,32)^

Food insecurity has been known to have characteristics of complexity and dynamic capacity. No income due to unemployment and reduce income because of sadden dismissal is increasing the risk for food insecurity.^(35)^ Based on previous studies, food insecure people, due to low income, consume high-fat, high-calorie food products which costs less than healthy foods.^(35)^ The increase energy and fat consumption may have a negative impact on an individual’s body weight leading to obesity^(36)^ and increasing the risk of non-communicable diseases such as cardiovascular diseases^(37)^ and type 2 diabetes.^(38)^ Moreover, food insecure people face an additional barrier in managing their chronic disease progression because of their inability to purchase foods indicated for their illness. For example, food insecure people with diabetes, reported poor glycemic control, as indicated by hemoglobin a(1c) ≥8.5%, due to the increase consumption of fat-rich foods and low intake of fruits and vegetables.^(38)^ In addition, low-income people with coeliac disease reported reduce adherence to gluten free diet due to its high cost and their low affordability in purchasing such a diet.^(24)^ As a result of food insecurity, multimorbidity may increase expenses for medications, transportation to doctors, and physiotherapy sessions.^(39)^ Based on the above, it seems that low income people with multimorbidity are in a high risk of being food insecure. However, the relationship between multimorbidity and food insecurity is vice versa, and more studies are needed for the identification of the real association and the effect of other confounding factors such as the socio-demographic characteristics.

Our results should be interpreted in the context of several limitations. First, the cross-sectional design allows us to report associations, which, however, have not casual nature. Furthermore, data collection through self-reporting increases the probability of recall bias. Moreover, the number of people that denied participation in the study was not considered. Also, using other sampling methods such as PPS (Probability-Proportional-to-Size sampling) we may have concluded to more accurate results. Lastly, food insecurity seasonal variations were not considered in the methodology. At the same time, the study has several strengths as it is a large population-based study using a representative sample of Cypriot population.

In conclusion, the prevalence of food insecurity in Cyprus is similar to that of other European countries. Moreover, our findings suggest that income is the strongest socio-demographic factor associated with food insecurity. Food insecurity is a global problem, affecting both developed and non-developed countries. The association between food insecurity and socio-demographic characteristics needs further examination in order for each country to develop specific public health policies targeted in specific population (e.g. financial support to low income people) so as to decrease food insecurity and improve people’s overall health and quality of life.
